# The Histaminergic System in Neuropsychiatric Disorders

**DOI:** 10.3390/biom11091345

**Published:** 2021-09-11

**Authors:** Li Cheng, Jiaying Liu, Zhong Chen

**Affiliations:** Key Laboratory of Neuropharmacology and Translational Medicine of Zhejiang Province, School of Pharmaceutical Sciences, Zhejiang Chinese Medical University, Hangzhou 310053, China; cheng_li@zju.edu.cn (L.C.); 18279727277@163.com (J.L.)

**Keywords:** histamine, histamine receptor, neuropsychiatric disorders, sleep disorders, schizophrenia, Alzheimer’s disease, Tourette’s syndrome, Parkinson’s disease

## Abstract

Histamine does not only modulate the immune response and inflammation, but also acts as a neurotransmitter in the mammalian brain. The histaminergic system plays a significant role in the maintenance of wakefulness, appetite regulation, cognition and arousal, which are severely affected in neuropsychiatric disorders. In this review, we first briefly describe the distribution of histaminergic neurons, histamine receptors and their intracellular pathways. Next, we comprehensively summarize recent experimental and clinical findings on the precise role of histaminergic system in neuropsychiatric disorders, including cell-type role and its circuit bases in narcolepsy, schizophrenia, Alzheimer’s disease, Tourette’s syndrome and Parkinson’s disease. Finally, we provide some perspectives on future research to illustrate the curative role of the histaminergic system in neuropsychiatric disorders.

## 1. Introduction

Neuropsychiatric disorders are a group of multifaceted diseases characterized by cognitive deficits, mental health symptoms and somatoform symptoms, including schizophrenia, depression, Alzheimer’s disease, Parkinson’s disease, etc. Neuropsychiatric disorders are chronic diseases beginning early in life and affecting patients across all age groups [1]. These conditions are a major public health challenge with highly prevalence, diminishing quality of life for millions of patients and their caregivers [2,3,4]. Although several approved treatments for neuropsychiatric disorders exist, the mechanisms are still incompletely understood and there are no highly-efficient therapeutic methods currently. Thus, it is urgent to illuminate the pathogenesis of neuropsychiatric disorders and develop new effective treatment methods.

It has been long known that histamine could trigger peripheral actions, such as allergic responses and gastric acid secretion via its receptors. However, the later extensive investigations discovered that histamine in the brain mediates diverse higher functions, including arousal, cognition and feeding, and have indicated the abnormalities in the histaminergic nervous system are closely related to neuropsychiatric disorders, including narcolepsy, schizophrenia, Alzheimer’s disease, Tourette’s syndrome and Parkinson’s disease. Since the histaminergic system is a recently discovered neuromodulatory system with anatomically differences, and histamine is a kind of relatively moderate neurotransmitter, the histaminergic system has garnered less attention than other neurotransmitter systems such as glutamatergic system and GABAergic system. In the past few decades, the knowledge of histamine neurobiology has continued to expand. Even though the numerous clinical trials have been disappointing, the H_3_R antagonist pitolisant has been approved for the treatment of narcolepsy. With the development of neuroscientific techniques, advanced studies of histaminergic system will provide potential drug target for the treatment of neuropsychiatric disorders.

## 2. Histaminergic Signaling in the Brain

### 2.1. Histamine in the Brain

In the brain, the histaminergic neuron bodies are concentrated in the tuberomammillary nucleus of the hypothalamus (TMN), and these neurons emerge late and mature slowly in the development of mammals [5,6]. TMN neurons are born at embryonic day 13–18 (E13–18), and intracellular histamine could be detected at embryonic day 20 (E20) in the rat [7]. The histamine synthesized by TMN neurons is stored in vesicles and released from the axon varicosities. There are about 4600 histaminergic neurons in the rat brain and about 64,000 histaminergic neurons in the human brain [6,8]. Most of these neurons have large somas (about 20–30 μm in diameter) and two to three relatively large multi-branched dendrites that often overlap each other and send fibers to almost all regions, including the cerebral cortex and the spinal cord [9]. The cerebral cortex, amygdala, substantia nigra (SN) and striatum receive moderate or dense histaminergic nerve terminals, while the projection density of hippocampus and thalamus varies among species [10]. The afferent projections to the TMN neurons are widely distributed in the brain, mainly including infralimbic cortex, lateral septum and preoptical nucleus [11]. It is reported that a subset of TMN histaminergic neurons also contain GABA and express the glutamic acid decarboxylase (GAD, GABA synthetic enzyme) [12,13,14]. Generally, the TMN histaminergic neurons exhibit low-frequency spontaneous firing rate (1–4 Hz) [9]. The firing rate of histaminergic neurons is higher during wakefulness than during sleep, resulting from inhibitory GABAergic inputs from the ventrolateral preoptic area (VLPO) [15,16]. In addition to histaminergic neurons, brain histamine is produced in mast cell, mainly present in the pia mater, thalamus and hypothalamus and the rate of histamine synthesis, release and metabolism in mast cells is much slower than in histaminergic neurons [7]. Moreover, microglia and ependymal cells in the brain may also produce histamine, but the role of these histamines has not been fully determined [15,17].

In mammals, histidine is brought into neurons by the L-amino-acid-transporter and then histamine is synthesized through catalyzing the oxidative decarboxylation of histidine by the rate-limiting enzyme histidine decarboxylase (HDC) [18]. The HDC activity in the TMN where the histaminergic neurons are located is high, as well as the histaminergic nerve endings [19]. After administration of α-FMH (α-fluoromethylhistidine, an irreversible and selective HDC inhibitor), the neuronal histamine is rapidly depleted within a few hours, while there is no obvious effect on mast cell-derived histamine [20]. Then the vesicular monoamine-transporter (VMAT) take up histamine into the vesicle storage. When the action potential arrives, histamine is released from the vesicle. In the absence of a high affinity uptake system for histamine, most of the released histamine is methylated by histamine N-methyltransferase (HNMT) located postsynaptically or in glia to inactive tele-methylhistamine (t-MH). Next, monoamine oxidases B (MAO-B) catalyze the oxidative deamination of t-MH to tele-methylimidazole acetic acid (t-MIAA) [10,21]. Due to its quit low activity in brain under physiological conditions, diamine oxidase (DAO) catabolizes histamine mainly in peripheral tissues. However, when the activity of HNMT is inhibited, DAO could metabolize histamine into imidazole acetaldehyde [9,22].

### 2.2. Histamine Receptors

There are mainly four known histamine receptor subtypes (H_1_R-H_4_R), all of which belong to the large family of G-protein-coupled receptors (GPCRs). A brief description of the histamine receptors is given in the following paragraphs.

#### 2.2.1. Histamine H_1_ Receptor (H_1_R)

Histamine H_1_ receptor (H_1_R) is widely distributed in the central nervous system, especially in the brain regions known to regulate arousal state and sleep-wakefulness, such as thalamus, cortex, cholinergic nuclei, locus coeruleus and raphe nucleus etc. [21,23]. The H_1_R (486-491 amino-acids) is encoded by a single structural gene on the long arm of chromosome 3. Intracellularly, the H_1_R is coupled to Gq/11 proteins and stimulates phospholipase C (PLC), resulting in the activation of neurons and astrocytes [24,25,26]. The activated PLC catalyzes the hydrolysis of phosphatidylinositol-4,5-biphosphate (PIP2) into two second messengers, inositol triphosphate (IP3) and diacylglycerol (DAG). IP3 induces the release of stored Ca^2+^ from intracellular stores into the cytoplasm and DAG mediates the activation of protein kinase C (PKC). Additionally, the activation of H_1_R also lead to the production of cyclic guanosine monophosphate(cGMP) and nitric oxide (NO) and increase the activity of phospholipase A2 (PLA2), which induces arachidonic acid formation [9,10].

#### 2.2.2. Histamine H_2_ Receptor (H_2_R)

Histamine H_2_ receptor (H_2_R) is mainly expressed in several brain areas including basal ganglia, hippocampus, amygdala and cerebral cortex [21,27]. The H_2_R (359 amino-acids) is located on the chromosome 5 and contains 8 exons. The H_2_R is coupled to Gs proteins and then stimulates adenylyl cylase, inducing an increase in intracellular cyclic adenosine monophosphate (cAMP) production. The increase in cAMP activates protein kinase A (PKA), which in turn phosphorylates its target proteins in the cytosol, cell membrane or translocate to the nucleus, and then activate the cAMP response element-binding protein (CREB). H_2_R activation also blocks a Ca^2+^-activated potassium conductance, inhibits PLA2 and release of arachidonic acid, which may explain why H_1_R and H_2_R have opposite physiological responses in many tissues [9,10].

#### 2.2.3. Histamine H_3_ Receptor (H_3_R)

Histamine H_3_ receptor (H_3_R) is widely distributed in the central nervous system, while low expressed in peripheral tissues. In situ hybridization studies reveal high levels of H_3_R mRNA in the cortex, hippocampus and caudate nucleus, followed by the anterior olfactory nucleus, amygdala, bed nucleus of stria terminalis, cerebellum and thalamus. The expression of H_3_R mRNA is low in habenula, zona incerta, globus pallidus, SN and substantia innominate etc. [28,29]. As a presynaptic autoreceptor on histaminergic neurons, the H_3_R mediates feedback inhibition of the release and synthesis of histamine. The H_3_R also distributes on the presynaptic membrane of non-histaminergic neurons and regulates the release of other neurotransmitters, such as dopamine, glutamate, GABA and acetylcholine. In addition, H_3_R acts as postsynaptic modulatory receptors in the striatum and cortex etc. [30]. The H_3_R (326–445 amino-acids) is located on chromosome 20q13.33. Due to the different connection between exons and introns, the coding region of the H_3_R gene can be composed of 3 exons (3965 bp) and 2 introns (2627 bp), or 4 exons (2418 bp) and 3 introns (2867 bp). The H_3_R is coupled to Gi/o proteins and plays an important role in the transduction process of downstream signaling pathways. The activation of H_3_R inhibits the adenylyl cyclase, decreasing the production of cAMP from adenosine triphosphate (ATP). H_3_R activation also leads to inhibition of high-voltage activated calcium channels, which reduces transmitter release in presynaptic terminals. In addition, H_3_R activates phosphorylation of the Akt/GSK-3 beta pathway, inwardly rectifying K+ channels, phospholipase C, phosphatidylinositide 3-kinases (PI3K) and mitogen-activated protein kinases (MAPK) [21,31,32].

#### 2.2.4. Histamine H_4_ Receptor (H_4_R)

Histamine H_4_ receptor (H_4_R) is recently identified as a new member of the histamine receptor family, which is mainly expressed on the cells of the hematopoietic lineage and immune cells, such as mast cells, eosinophils and dendritic cells. H_4_R is also reported present in microglia with unconvincing evidence, whose function is still unclear. At present, the research on H_4_R mainly focuses on its role in the inflammatory process mediated by histamine. The H_4_R (390 amino-acids) is located on chromosome *18q11.2* and contains 3 exons and 2 introns. Moreover, H_4_R reveals ~40% homology with H_3_R and acts through Gi/o proteins to reduce the accumulation of cAMP. In addition to cAMP, Ca^2+^ is also the second messenger downstream of the H_4_R. Activation of H_4_R also increases the accumulation of Ca^2+^, activates the kinases (PI3K, MAPK, ERK) and transcription factor activator protein-1. In addition, H_4_R can bind to β-arrestin to activate MAPK pathways.

## 3. The Histaminergic System in Neuropsychiatric Disorders

The histaminergic system plays an important role in regulating various functions of the brain, such as sleep and wakefulness, learning and memory, feeding and energy balance. This review mainly introduces preclinical and clinical studies exploring the potential role of histaminergic system in neuropsychiatric disorders, including narcolepsy, schizophrenia, Alzheimer’s disease, Tourette’s syndrome and Parkinson’s disease. Currently, several histamine receptor ligands are in clinical trials for the potential treatment of these neuropsychiatric disorders (Table 1).

### 3.1. Sleep Disorders

**Figure 1:**
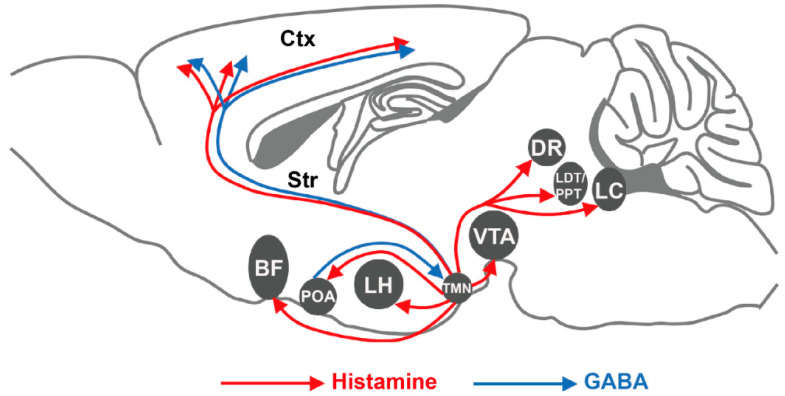
A schematic diagram of circuits regulating the sleep and wakefulness by the TMN histaminergic system. BF, basal forebrain; Ctx, cortex; DR, dorsal raphe; LC, locus coeruleus; LDT/PPT, laterodorsal/pedunculopontine tegmental nuclei; LH, lateral hypothalamic; POA, preoptic area; Str, striatum; TMN, tuberomammillary nucleus; VTA, ventral tegmental area.

Histamine system is strongly suggested to play an essential role in modulating sleep and wake behavior via H_1_R and/or H_3_R. Histamine release is found to have a circadian rhythm, which is responsible for the modulation of sleep and wakefulness [33,34]. The histaminergic TMN neurons fire only during wakefulness and their activities are related to a high level of vigilance. In contrast, they cease firing and then remain quiescent during slow-wave sleep (SWS) and rapid eye movement sleep (REM) [16]. Additionally, the expression of immediate-early gene c-fos (a maker of neuronal activation) in histaminergic TMN neurons is higher during periods of wakefulness [23,35]. These results demonstrate that histaminergic neurons are wake-active and may play a crucial role in regulating wakefulness and wake-related behaviors. To elucidate the functions of histamine system on sleep-wakefulness, the genetically knockout mice are generated and has boosted research on histamine powerfully [36,37,38,39]. Compared to wild-type mice, *HDC^−/−^* mice exhibit a fragmented sleep-wake architecture with shortened episode duration and increased frequency of episodes in wakefulness and SWS, an increased REM sleep episodes mainly during the light phase and no major change in the daily amount of wakefulness or SWS [40]. *Hrh1^−/−^* mice have duration and circadian profile of sleep and wakefulness basically the same as wild type mice with exceptions of reduced number of brief awakenings, prolonged duration of SWS and fewer transitions between SWS and wakefulness [41]. H_1_R is also expressed in astrocytes and astrocytic H_1_R regulates circadian rhythms and quality of wakefulness, but not the quantity and quality of sleep [25]. Interestingly, *Hrh3^−/−^* mice show signs of increased histamine transmission and vigilance, while they exhibit deficient wakefulness and sleep fragmentation, which is most likely due to the decreased activity of histaminergic neurons and desensitization of postsynaptic histaminergic receptors [42]. Furthermore, numerous pharmacological interventions have been reported with similar conclusions [43,44,45]. At present, research into the neural circuitry of sleep and wakefulness has made remarkable progress. The VLPO of the hypothalamus is essential for sleep regulation and anatomical studies have shown that the VLPO and TMN are connected. The release of histamine from TMN neurons by using photostimulation disinhibits the wake-active TMN neurons and indirectly suppresses sleep-active VLPO neurons through the activation of the GABAergic interneuron [46]. A recent study found that the preoptic area GABAergic neurons projecting to the TMN are both sleep active and sleep promoting by using a lentivirus for retrograde labelling and optogenetic manipulation [47]. In addition, the orexin/hypocretin (hypocretin neuropeptide precursor, HCRT) neurons are one important input to innervate and excite the TMN neurons during wakefulness [48,49]. Infusion of orexin A produces a significant increase in wakefulness, which dependeds on the activation of histaminergic neurotransmission mediated by H_1_R [50]. On the other hand, histamine also plays a regulatory role on the developing HCRT system via H_1_R [51]. It has been suggested that activity of TMN histaminergic neurons is important for enhancing arousal under certain conditions, such as exposure to a novel environment. Given the correlation between TMN histaminergic neurons excitability and behavioral arousal, Fujita et al. found that the tonic firing of histaminergic neurons is necessary for the maintenance of arousal during wakefulness, and their silencing is sufficient to impair arousal and induce SWS rapidly and selectively [52,53]. Additionally, some histamine neurons have the capacity to express the glutamic acid decarboxylase-67 (GAD67, GABA-synthesizing enzyme) and the vesicular GABA transporter (Vgat) gene [12,54]. Abdurakhmanova et al. show nice double-label in situ hybridization of HDC, Gad67 and Vgat mRNAs in the TMN and find that both histamine and GABA, released from histamine/GABA neurons, are involved in regulation of brain arousal states [55]. Selective deletion of Vgat gene expression from histaminergic TMN neurons increased wake during the night and locomotion [12]. A recent single-cell RNA sequencing (scRNA-seq) study found that TMN histaminergic neurons co-express very low Slc32a1 (encoding the vesicular GABA transporter) and exhibit a degree of transcriptional heterogeneity, a finding that adds further complexity to the heterogenous functions of these neurons [56]. Given the heterogeneity of TMN histaminergic neurons, it could be that the subset of histamine/GABA neurons these go into the cortex/striatum have substantial influence via volume transmission and can allow GABA release, and yet other populations (projecting to the VLPO) do not co-express GABA [52]. Further, the TMN histaminergic neurons innervate various downstream regions to regulate sleep-wakefulness, such as the cholinergic neurons in the basal forebrain (BF) or pedunculopontine and laterodorsal tegmentum, the serotonergic neurons in the dorsal raphe nucleus (DR), the dopaminergic neurons in the ventral tegmental area (VTA) or the noradrenergic neurons in the locus coeruleus (LC) (Figure 1) [23,57,58].

Narcolepsy is a disabling and chronic neurological disorder primarily characterized by irresistible sleep episodes and cataplexy [59]. Given histaminergic system comprises a major component of the arousal system and regulates sleep-wake cycle, its effect on narcolepsy has been extensively studied. Several studies have confirmed reduced cerebrospinal fluid (CSF) histamine levels in human narcolepsy especially in hypocretin-deficient narcolepsy [60,61,62], while a recent study reported that narcoleptic children with hypocretin deficiency had a higher CSF histamine level together with a lower t-MH level leading to a decreased histamine turnover and an impairment of histaminergic neurotransmission [63]. In addition, the number of histaminergic TMN neurons increased in patients with narcolepsy compared with the control group [64,65]. However, Robert et al. found that there were no significant differences between narcoleptic patients and control subjects in CSF histamine or t-MH levels [66,67]. Taken together, these above observations indicate the histaminergic system changes in the brain of narcoleptic patients, but further research is needed, especially the role of histaminergic system in different phenotypes of narcolepsy. The H_3_R antagonist/inverse agonist pitolisant (formerly known as BF2.649; tiprolisant) has been approved in the EU for the treatment of narcolepsy with or without cataplexy in adult patients and in the USA for the treatment of excessive daytime sleepiness in adult patients with narcolepsy [68]. Additionally, clinical studies have confirmed the long-term safety and therapeutic effect of pitolisant on daytime sleepiness, cataplexy, hallucinations and sleep paralysis repeatedly [69,70] (Table 1). Further, pitolisant is generally well tolerated and the severity of most adverse events are mild or moderate. Thus, based on the above findings, pitolisant could constitute an alternative treatment for patients with narcolepsy [68]. In summary, the TMN histaminergic neurons itself or through the innervation of other nervous systems participates in regulating the development of narcolepsy, and it is a potential target worth exploring for narcolepsy treatment. More importantly, the detailed mechanisms still need to be answered.

### 3.2. Schizophrenia

Schizophrenia is a common and severe psychiatric syndrome characterized by positive symptoms (e.g., delusions, hallucinations and paranoia), negative symptoms (e.g., alogia, social withdrawal, flattened affect and anhedonia) and cognitive deficits (impaired executive function, working memory and processing speed). Schizophrenia affects approximately 1% of the world population and causes considerable distress to the individual and society [71,72,73]. In recent years the role of histamine or its receptor as a pathophysiological contributor to a range of neuropsychiatric disorders, such as schizophrenia, has attracted the attention of researchers. Post-mortem studies have shown that the mean level of t-MH was elevated in the CSF of schizophrenia patients, suggesting increased central histaminergic activity in these patients [74]. In the dorsolateral prefrontal cortex, the average H_3_R expression of the schizophrenia patients, especially the ones treated with atypical antipsychotics, was significantly higher than those of the controls [75]. However, the human positron emission tomography (PET) study showed lower H_1_R binding in the frontal cortex, prefrontal cortex and cingulate cortex of schizophrenia people [76]. Indeed, some second-generation antipsychotics, including clozapine and olanzapine, have potent antagonistic effects on H_1_R. However, it remains incompletely understood that the ability of antipsychotics to block H_1_R is responsible for the therapeutic effects or the side effects of these compounds. Accordingly, the relationships between H_1_R occupancy and the main and side effects of atypical antipsychotics in patients should be elucidated in future [77]. Clinical trials have suggested the important influence of histamine receptors on schizophrenia patients, though the data are mixed. Since randomized clinical trial in 1990 of a positive therapeutic effect of the H_2_R antagonist famotidine on the negative symptoms in schizophrenia, the open-label study also indicated the effective role of the H_2_R antagonist ranitidine in negative symptoms [78,79]. Moreover, in a placebo-controlled, randomized clinical trial, famotidine has been observed to be beneficial to both the positive and negative symptoms in treatment-resistant schizophrenia, implying that H_2_R antagonism may provide a new alternative for the treatment of schizophrenia. Even though the preclinical studies in pharmacological models of schizophrenia have shown the protective effects of H_3_R inverse agonists, such as ABT-239, pitolisant, GSK207040, on the locomotor hyperactivity, the cognitive and sensory gating deficits, the clinical results of H_3_R inverse agonists in schizophrenia were disappointing unfortunately [80,81,82]. It has been reported that the H_3_R inverse agonists MK0249, ABT-288 and GSK239512, were not superior to placebo in the treatment of cognitive impairment in schizophrenia patients (Table 1). Further, the antipsychotics are widely used in the treatment of schizophrenia and one of the common adverse effects is weight gain, which is associated with increased risk of obesity in patients with schizophrenia. Betahistine, a weak partial H_1_R agonist and H_3_R antagonist, seem to reduce weight gain caused by antipsychotic drugs [83,84].

**Figure 2:**
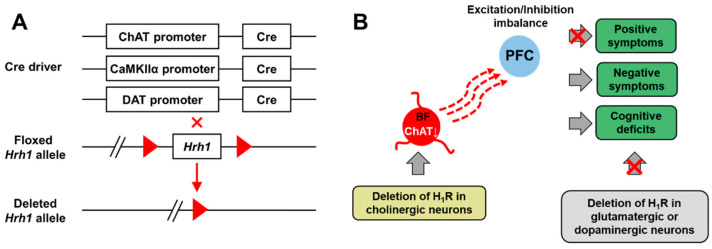
H_1_R in basal forebrain (BF) cholinergic neurons plays a critical role in the pathogenesis of negative symptoms in schizophrenia. (**A**) Schematic diagram of the generation of conditional knockout mice. (**B**) Schematic diagram of the working model [87]. BF, basal forebrain; PFC, prefrontal cortex.

Our previous study indicates that H_1_R plays a cell-type-specific role in the brain [85,86]. Accordingly, we hypothesize that the cell-type specific H_1_R may be involved in the pathogenesis of schizophrenia. In our recent study, we generate the mice with a targeted deletion of H_1_R in different types of neurons, including glutamatergic neurons (*CaMKIIα-Cre;Hrh1^fl/fl^*), dopaminergic neurons (*DAT-Cre;Hrh1^fl/fl^*) or cholinergic neurons (*ChAT-Cre;Hrh1^fl/fl^*) by using the Cre-LoxP system and find that *ChAT-Cre;Hrh1^fl/fl^* mice, instead of *CaMKIIα-Cre;Hrh1^fl/fl^* or *DAT-Cre;Hrh1^fl/fl^* mice, exhibit the behavioral deficits related to negative symptoms of schizophrenia. Then we confirm that the H_1_R expression in cholinergic neurons of basal forebrain (BF) is significantly decreased in patients with schizophrenia having negative symptoms. Finally, we verify that H_1_R in BF cholinergic neurons plays a key role in the pathogenesis of behavioral deficits in *ChAT-Cre;Hrh1^fl/fl^* mice and identify the underlying circuit mechanism by selective re-expressing H_1_R in BF cholinergic neurons and activating/inhibiting the BF cholinergic neurons with chemogenetic methods (Figure 2) [87]. Our results suggest cell-type and BF region specific depletion of H_1_R functionality is responsible for the pathogenesis of negative symptoms of schizophrenia, which may be useful for the development of new drugs specifically aimed at patients expressing predominantly negative symptoms. Our study suggests that the complex results of previous postmortem sample studies and clinical trials may be due to the lack of selective interventions for brain regions and cell types. Clinical therapy for schizophrenia would benefit from the development of effective drug carrier for histamine receptor-targeted drug specifically delivered to brain regions or cell types.

### 3.3. Alzheimer’s Disease (AD)

Alzheimer’s disease (AD) is a slowly progressive neurodegenerative disease and is marked by progressive memory loss, language impairment, behavioral changes and loss of functional abilities. AD is the most common cause for dementia and is characterized by the neurodegeneration, the loss of synapses, extracellular amyloid β (Aβ) peptide-containing neuritic plaques and intracellular tau-positive neurofibrillary tangles in the most selected regions of the brain [88,89,90]. The role of histaminergic system in AD has remained conflicting. In 1989, Mazurkiewicz-Kwilecki and colleagues observed that the histamine levels in the frontal, temporal and occipital cortices and the caudate nucleus of postmortem AD samples were statistically significant decreased by using the double isotope technique [91], whereas Cacabelos and colleagues reported that the histamine levels in numerous regions except for the corpus callosum and globus pallidus, such as temporal cortex, hippocampus, putamen, caudate nucleus, thalamus and hypothalamus, were significantly higher in AD patients than controls by using the high-performance liquid chromatography (HPLC) with fluorometric detection [92]. Considering the limitations of past technology, Panula et al. used a very sensitive HPLC method in 1998, and found that the histamine content was significantly reduced in the hypothalamus, hippocampus and temporal cortex and showed no obvious change in other cortical areas, putamen and SN of AD samples [93]. Furthermore, subsequent studies confirmed that the total number of TMN neurons was significantly (57%) lost in AD patients, while in contrast there was no significant (24%) difference of total HDC mRNA expression in the entire TMN between AD patients and controls, suggesting compensatory processes [94,95]. It should be noted that H_3_R- and HNMT-mRNA expression in the prefrontal cortex increased only in female AD patients [94]. Accordingly, these discrepant findings may be attributable to complex factors, such as gender, age, postmortem delay (PMD), post mortem storage times and temperatures. Further, the amount of H_1_R binding assessed by positron emission tomography was significantly reduced in the frontal and temporal areas of AD patients compared to controls, revealing a disruption of the histaminergic neurotransmission in AD pathology [96].

There is a vast literature to unravel the essential role of histaminergic system in several aspect of fear and recognition memory acquisition, consolidation and retrieval, whose impairment is the first and most prominent symptom of AD [97,98]. The knockout mice are applied to explore the influence of the histaminergic system on cognition and the behavioral results of most knockout mice indicate signs of impaired learning and memory. Both *Hrh1^−/−^* and *Hrh2^−/−^* mice show impaired object recognition and spatial learning and hippocampal long-term potentiation (LTP), but improved acquisition of auditory and contextual freezing [99]. *Hrh1^−/−^* mice also exhibit impairments of spatial reference and working memory in a reward-driven eight-arm radial maze task, temporal object memory and long-term motor memory [100,101,102]. In contrast to *Hrh1^−/−^* mice that show unchanged performance in the passive avoidance test, *Hrh2^−/−^* mice, compared to wild type mice, take more time to enter the dark compartment associated with electric shock [103,104]. Pharmacologic experiments also demonstrate that H_1_R antagonist impairs object recognition memory, spatial cognition in eight-arm radial maze and inhibitory avoidance memory retrieval [105,106] and H_2_R antagonist impairs object recognition memory and inhibitory avoidance memory [105]. However, contradictory results are also reported (e.g., intraventricular infusion of the H_1_R antagonist chlorpheniramine improves the performance in Morris water maze) [107]. Together, the above studies indicate that H_1_R or H_2_R has important impacts on the learning and memory implying that histaminergic system may be involved in the regulation of cognitive dysfunction in AD. Since HDC and H_3_R have unspecific effects on the production and release of histamine or several other neurotransmitters, *HDC^−/−^* and *Hrh3^−/−^* mice exhibit more complex behavior changes. Similar to *Hrh1^−/−^* and *Hrh2^−/−^* mice, *HDC^−/−^* mice have improved auditory and contextual freezing, but they show no difference with wild type mice in novel object recognition test and perform better in the hidden platform water maze test [108,109,110,111,112]. H_3_R-deficiency do not seem to affect memory in object recognition and passive avoidance test, while the deficiency is associated with improved spatial learning and memory in the Barnes maze [39,113]. H_3_R antagonists show improved cognition in eight-arm radial maze task, water maze, Y-maze and so on [114,115,116]. In addition, a similar procognitive effect is observed when the APPTg2576 AD transgenic mice administered with H_3_R antagonist ciproxifan could alleviate the discrimination deficits in the object recognition test [117]. On the basis of the preclinical findings, a series of clinical trials on H_3_R antagonists for the treatment of AD have been carried out. In a double-blind, randomized, placebo controlled, parallel group study with a small group of patients (*n* = 8), over 4 weeks treatment of GSK239512 (a potent and selective H_3_R antagonist) displays a satisfactory level of tolerability and improved cognitive function in AD patients with mild to moderate symptoms [118]. In a subsequent randomized, double-blind, placebo-controlled, 16-week study using a larger population, GSK239512 is used as a monotherapy in subjects with mild-to-moderate AD and improved episodic memory, but not executive function/working memory or other domains of cognition [119]. A randomized study of H_3_R antagonist ABT-288 to evaluate its efficacy and safety in subjects with mild-to-moderate AD is prematurely terminated, because ABT-288 dose groups do not significantly differ from placebo group, while the active comparator donepezil demonstrates statistically significant improvement, suggesting ABT-288 shows no efficacy in the symptomatic treatment of AD [120]. Another H_3_R inverse agonist MK0249 obtains similar results that administration of MK0249 over 4 weeks has no effect on cognitive function in mild to moderate AD patients [121] (Table 1). Even though these clinical trials fail to demonstrate unequivocal cognitive improvements, the first H_3_R antagonist pitolisant has received market approval from the European Medicines Agency for the treatment of narcolepsy. Thus, the procognitive activity of pitolisant may also expand therapeutic applications in AD.

### 3.4. Tourette’s Syndrome (TS)

Tourette’s syndrome (TS) is a developmental neuropsychiatric disorder characterized by multiple motor and vocal tics, present in childhood and lasting more than one year. According to the DSM-5 definition, tics are “sudden, rapid, recurrent, nonrhythmic motor movements or vocalizations, generally preceded by urge” [122,123]. There have been many investigations into the relationship between the neuronal histaminergic system dysregulation and TS over the years. In 2010, Ercan-Sencicek and colleagues identified a nonsense mutation (W317X) in the HDC gene encoding L-histidine decarboxylase by an analysis of linkage in a two-generation family pedigree with an extremely high frequency of TS and represented that histamine dysregulation was related to TS for the first time [124]. Subsequently, Fernandez and Karagiannidis et al. studied variation across HDC for association with TS beyond this single family in succession and further supported the histaminergic hypothesis in TS etiology [125,126]. These genetic findings strongly implicate a causal relationship between the HDC mutations and TS and suggested a role for histaminergic system in the mechanism and modulation of TS. Accordingly, a number of studies have examined the *HDC^−/−^* mice in a variety of contexts especially the TS and related conditions. At baseline, *HDC^−/−^* mice exhibit no tic-like movements, elevated rearing or evident spontaneous motor stereotypies. However, *HDC^−/−^* mice show markedly increased motor locomotion and stereotypies after the acute administration with psychostimulant D-amphetamine when compared to wild type mice, suggesting that *HDC* deficiency can potentiate tics and tic-like stereotypies. Moreover, the stereotypies in *HDC^−/−^* mice could be mitigated by pretreatment with an efficacious treatment for tics, the D_2_R antagonist haloperidol. Another kind of behavioral defect of sensorimotor gating, which is reflected in a deficit in prepulse inhibition can be measured in TS patients carrying the HDC W317X mutation and the *HDC^−/−^* mice, which provided an additional behavioral parallel between TS patients and the *HDC^−/−^* mouse model. The pathophysiological mechanisms in the *HDC^−/−^* mouse model may be that reduced histamine production result in dysregulation of dopaminergic modulation of the basal ganglia and produces TS phenomenology [127]. Further, the centrality of striatal regulation by histaminergic TMN neurons is reported to be responsible for the production of pathological grooming and the H_3_R expression in the striatum of *HDC^−/−^* mice is markedly up-regulated, suggesting the H_3_R in the striatum is a contributor to the pathology and emerges as a novel treatment of tic disorders [128,129]. It has been reported that a male patient with tics and narcolepsy is treated with H_3_R antagonist/inverse agonist pitolisant and his daytime sleepiness decreased dramatically, whereas tic scores remain constant. Additionally, a two-part, randomized, multi-center, blinded study of H_3_R antagonist AZD5213 in adolescents with TS is conducted (Table 1). Compared to placebo group, the subjects exhibit no statistically difference when treated with the lower dose of drug and more severe tic symptoms when treated with the higher dose of drug. This worsening of tics may be interpreted as confirming the relevance of the receptor to pathophysiology of TS; however, the details need further investigations. Thus, more work is awaited to confirm the histaminergic hypothesis in TS to provide new strategies for disease treatment and prevention.

### 3.5. Parkinson’s Disease (PD)

Parkinson’s disease (PD) is a progressive neurodegenerative disorder characterized by motor symptoms (i.e., resting tremor, rigidity, bradykinesia and postural instability) and non-motor symptoms (i.e., dementia, hyposmia and gastrointestinal alterations). PD is commonly accompanied by the loss of dopaminergic neurons in the substantia nigra pars compacta (SNc), the major cause of Parkinsonian motor symptoms viz, and widespread presence of α-synuclein aggregations in the form of Lewy bodies (LBs) or Lewy neurites (LNs) [130,131]. Conflicting findings about the role of the neuronal histaminergic system in PD have been reported. Post mortem brain tissues of patients with PD show a strong accumulation of LBs and LNs in the TMN, indicating that TMN is severely damaged in the course of PD [132]. In spite of the abnormal accumulation in the TMN, no significant difference of HDC mRNA levels and number of histaminergic neurons are observed in TMN between PD patients and controls [133,134]. However, Rinne and colleagues found that although t-MH concentrations were unchanged in the putamen and temporal cortex, the local histamine levels of patients with PD significantly were increased in the SN (201%), putamen (159%) and globus pallidus (234%), who are responsible for motor behavior and functional changes in PD [135]. This is in line with the reports that in brains with PD the density of histaminergic fibers in the SN is increased, the morphology of histaminergic fibers is thinner than that of the control group and the varicosities are enlarged [136]. Furthermore, in the SN of PD patients the H_3_R mRNA expression is significantly reduced and HNMT mRNA expression is increased, and the level of HNMT mRNA correlate with PD disease duration negatively [137]. Together, the above observations imply although histamine production in the TMN does not alter significantly in PD, the local changes in the areas innervated by histaminergic neurons may contribute to PD pathology. It’s noteworthy that local histamine changes in the areas, especially the SN, may be a rationale for potential therapeutic strategies.

In addition to post-mortem sample research, animal models and pharmacological research are used to study the pathogenesis of PD. The injection of α-FMH could decrease the rotation behavior induced by apomorphine and prevented the loss of Tyrosine hydroxylase (a marker for dopaminergic neurons) expressing cells in the 6-hydroxydopamine (6-OHDA)-lesioned rat, which is a classic PD model [138]. Moreover, HDC, H_1_R/H_2_R antagonists, and H_3_R agonist reduce the apomorphine-induced turning behavior in the 6-OHDA-lesioned rat [139]. H_2_R antagonist ranitidine and famotidine reduce dyskinesia induced by levodopa in rat models of PD [140,141]. Further, the histamine level is significantly increased and apomorphine-induced behavioral response is mainly alleviated by H_3_R antagonist thioperamide in 6-OHDA-lesioned rats [142]. Thioperamide also could rescue the memory impairment in the mouse model of PD [143]. The above findings imply that the histaminergic system acts as a modulating role in rats lesioned to model PD and may provide new drug therapies for PD. The clinical study show that H_2_R antagonist famotidine eases bradyphrenia and improve motor function in patients with PD [144]. In a single-blind trial of PD patients with excessive daytime sleepiness, the H_3_R antagonist pitolisant alleviates excessive sleepiness, but the motor performance is not significantly affected [145]. Nevertheless, these such compounds increasing local release of histamine in specific brain regions may be promising and provide therapeutic insights for PD treatment, which needs further exploration.

## 4. Concluding Remarks and Future Prospects

There is plethora of evidence implicating histaminergic system to play an essential role in the regulation of neuropsychiatric disorders. However, many challenges are still existing that need large amount of the future research: (1) to identify the precise and detailed mechanisms by which histaminergic system acts to regulate the neuropsychiatric disorders, (2) to elucidate the brain region—and cell-type specific role of histamine receptors and (3) to create selective and specific histamine receptors ligands that can be used to treat neuropsychiatric disorders. It is promising that further studies shed light on mechanism and pathology of neuropsychiatric disorders by using cutting-edge technology, such as chemo-genetic and optogenetic approaches, and facilitate the development of new drugs targeting the histaminergic system for the treatment of neuropsychiatric disorders.

## Figures and Tables

**Table 1:** Histamine receptors ligands in clinical trials for the treatment of neuropsychiatric disorders.

Disorder	Ligands	Target	Phase	NCT Number
Narcolepsy	TS-091	H_3_R inverse agonist/antagonist	II	NCT03267303
	GSK189254	H_3_R inverse agonist/antagonist	II	NCT00366080
	JNJ-17216498	H_3_R antagonist	II	NCT00424931
	BF2.649	H_3_R inverse agonist/antagonist	III	NCT01638403
	BF2.649	H_3_R inverse agonist/antagonist	III	NCT01399606
	BF2.649	H_3_R inverse agonist/antagonist	III	NCT01067222
	BF2.649	H_3_R inverse agonist/antagonist	III	NCT01800045
	PF-03654746	H_3_R antagonist	II	NCT01006122
Schizophrenia	Famotidine	H_2_R antagonist	IV	NCT00565175
	BF2.649	H_3_R inverse agonist/antagonist	II	NCT00690274
	GSK239512	H_3_R inverse agonist/antagonist	II	NCT01009060
	MK0249	H_3_R inverse agonist/antagonist	II	NCT00506077
	ABT-288	H_3_R inverse agonist/antagonist	II	NCT01077700
Alzheimer’s disease	GSK239512	H_3_R inverse agonist/antagonist	II	NCT01009255
	MK0249	H_3_R inverse agonist/antagonist	II	NCT00420420
	PF-03654746	H_3_R antagonist	I	NCT01028911
	ABT-288	H_3_R inverse agonist/antagonist	II	NCT01018875
Tourette’s syndrome	AZD5213	H_3_R inverse agonist/antagonist	II	NCT01904773
Parkinson’s disease	Famotidine	H_2_R antagonist	II	NCT01937078
	BF2.649	H_3_R inverse agonist/antagonist	III	NCT01036139
	BF2.649	H_3_R inverse agonist/antagonist	III	NCT01066442

## References

[1] Kessler R.C., Amminger G.P., Aguilar-Gaxiola S., Alonso J., Lee S., Ustün T.B. (2007). Age of onset of mental disorders: A review of recent literature. Curr. Opin. Psychiatry.

[2] Kessler R.C., Berglund P., Demler O., Jin R., Merikangas K.R., Walters E.E. (2005). Lifetime prevalence and age-of-onset distributions of DSM-IV disorders in the National Comorbidity Survey Replication. Arch. Gen. Psychiatry.

[3] Fineberg N.A., Haddad P.M., Carpenter L., Gannon B., Sharpe R., Young A.H., Joyce E., Rowe J., Wellsted D., Nutt D.J. (2013). The size, burden and cost of disorders of the brain in the UK. J. Psychopharmacol..

[4] Gooch C.L., Pracht E., Borenstein A.R. (2017). The burden of neurological disease in the United States: A summary report and call to action. Ann. Neurol..

[5] Schwartz J.C., Arrang J.M., Garbarg M., Pollard H., Ruat M. (1991). Histaminergic transmission in the mammalian brain. Physiol. Rev..

[6] Panula P., Nuutinen S. (2013). The histaminergic network in the brain: Basic organization and role in disease. Nat. Rev. Neurosci..

[7] Panula P., Sundvik M., Karlstedt K. (2014). Developmental roles of brain histamine. Trends Neurosci..

[8] Baronio D., Gonchoroski T., Castro K., Zanatta G., Gottfried C., Riesgo R. (2014). Histaminergic system in brain disorders: Lessons from the translational approach and future perspectives. Ann. Gen. Psychiatry.

[9] Haas H.L., Sergeeva O.A., Selbach O. (2008). Histamine in the nervous system. Physiol. Rev..

[10] (2003). Haas H and Panula P, The role of histamine and the tuberomamillary nucleus in the nervous system. Nat. Rev. Neurosci..

[11] Ericson H., Blomqvist A., Köhler C. (1991). Origin of neuronal inputs to the region of the tuberomammillary nucleus of the rat brain. J. Comp. Neurol..

[12] Yu X., Ye Z., Houston C.M., Zecharia A.Y., Ma Y., Zhang Z., Uygun D.S., Parker S., Vyssotski A.L., Yustos R. (2015). Wakefulness Is Governed by GABA and Histamine Cotransmission. Neuron.

[13] Kukko-Lukjanov T.K., Panula P. (2003). Subcellular distribution of histamine, GABA and galanin in tuberomamillary neurons in vitro. J. Chem. Neuroanat..

[14] Trottier S., Chotard C., Traiffort E., Unmehopa U., Fisser B., Swaab D.F., Schwartz J.C. (2002). Co-localization of histamine with GABA but not with galanin in the human tuberomamillary nucleus. Brain Res..

[15] Brown R.E., Stevens D.R., Haas H.L. (2001). The physiology of brain histamine. Prog. Neurobiol..

[16] Takahashi K., Lin J.S., Sakai K. (2006). Neuronal activity of histaminergic tuberomammillary neurons during wake-sleep states in the mouse. J. Neurosci..

[17] Katoh Y., Niimi M., Yamamoto Y., Kawamura T., Morimoto-Ishizuka T., Sawada M., Takemori H., Yamatodani A. (2001). Histamine production by cultured microglial cells of the mouse. Neurosci. Lett..

[18] Huang H., Li Y., Liang J., Finkelman F.D. (2018). Molecular Regulation of Histamine Synthesis. Front Immunol..

[19] Nuutinen S., Panula P. (2010). Histamine in neurotransmission and brain diseases. Adv. Exp. Med. Biol..

[20] Slotkin T.A., Slepetis R.J., Weigel S.J., Whitmore W.L. (1983). Effects of alpha-fluoromethylhistidine (FMH), an irreversible inhibitor of histidine decarboxylase, on development of brain histamine and catecholamine systems in the neonatal rat. Life Sci..

[21] Hu W., Chen Z. (2017). The roles of histamine and its receptor ligands in central nervous system disorders: An update. Pharmacol. Ther..

[22] Kitanaka J., Kitanaka N., Tsujimura T., Terada N., Takemura M. (2002). Expression of diamine oxidase (histaminase) in guinea-pig tissues. Eur. J. Pharmacol..

[23] Yoshikawa T., Nakamura T., Yanai K. (2021). Histaminergic neurons in the tuberomammillary nucleus as a control centre for wakefulness. Br. J. Pharmacol..

[24] Panula P., Chazot P.L., Cowart M., Gutzmer R., Leurs R., Liu W.L., Stark H., Thurmond R.L., Haas H.L. (2015). International Union of Basic and Clinical Pharmacology. XCVIII. Histamine Receptors. Pharmacol. Rev..

[25] Kárpáti A., Yoshikawa T., Naganuma F., Matsuzawa T., Kitano H., Yamada Y., Yokoyama M., Futatsugi A., Mikoshiba K., Yanai K. (2019). Histamine H(1) receptor on astrocytes and neurons controls distinct aspects of mouse behaviour. Sci. Rep..

[26] Kárpáti A., Yoshikawa T., Nakamura T., Iida T., Matsuzawa T., Kitano H., Harada R., Yanai K. (2018). Histamine elicits glutamate release from cultured astrocytes. J. Pharmacol. Sci..

[27] Karlstedt K., Senkas A., Ahman M., Panula P. (2001). Regional expression of the histamine H(2) receptor in adult and developing rat brain. Neuroscience.

[28] Pillot C., Heron A., Cochois V., Tardivel-Lacombe J., Ligneau X., Schwartz J.C., Arrang J.M. (2002). A detailed mapping of the histamine H(3) receptor and its gene transcripts in rat brain. Neuroscience.

[29] Sallmen T., Lozada A.F., Anichtchik O.V., Beckman A.L., Panula P. (2003). Increased brain histamine H3 receptor expression during hibernation in golden-mantled ground squirrels. BMC Neurosci..

[30] Ellenbroek B.A., Ghiabi B. (2014). The other side of the histamine H3 receptor. Trends Neurosci..

[31] Nieto-Alamilla G., Márquez-Gómez R., García-Gálvez A.M., Morales-Figueroa G.E., Arias-Montaño J.A. (2016). The Histamine H3 Receptor: Structure, Pharmacology, and Function. Mol. Pharmacol..

[32] Mariottini C., Scartabelli T., Bongers G., Arrigucci S., Nosi D., Leurs R., Chiarugi A., Blandina P., Pellegrini-Giampietro D.E., Passani M.B. (2009). Activation of the histaminergic H3 receptor induces phosphorylation of the Akt/GSK-3 beta pathway in cultured cortical neurons and protects against neurotoxic insults. J. Neurochem..

[33] Orr E., Quay W.B. (1975). Hypothalamic 24-h rhythms in histamine, histidine, decarboxylase and histamine-N-methyltransferase. Endocrinology.

[34] Friedman A.H., Walker C.A. (1968). Circadian rhythms in rat mid-brain and caudate nucleus biogenic amine levels. J. Physiol..

[35] Ko E.M., Estabrooke I.V., McCarthy M., Scammell T.E. (2003). Wake-related activity of tuberomammillary neurons in rats. Brain Res..

[36] Watanabe T., Yanai K. (2001). Studies on functional roles of the histaminergic neuron system by using pharmacological agents, knockout mice and positron emission tomography. Tohoku J. Exp. Med..

[37] Inoue I., Yanai K., Kitamura D., Taniuchi I., Kobayashi T., Niimura K., Watanabe T., Watanabe T. (1996). Impaired locomotor activity and exploratory behavior in mice lacking histamine H1 receptors. Proc. Natl. Acad. Sci. USA.

[38] Takahashi K., Suwa H., Ishikawa T., Kotani H. (2002). Targeted disruption of H3 receptors results in changes in brain histamine tone leading to an obese phenotype. J. Clin. Investig..

[39] Toyota H., Dugovic C., Koehl M., Laposky A.D., Weber C., Ngo K., Wu Y., Lee D.H., Yanai K., Sakurai E. (2002). Behavioral characterization of mice lacking histamine H(3) receptors. Mol. Pharmacol..

[40] Parmentier R., Ohtsu H., Djebbara-Hannas Z., Valatx J.L., Watanabe T., Lin J.S. (2002). Anatomical, physiological, and pharmacological characteristics of histidine decarboxylase knock-out mice: Evidence for the role of brain histamine in behavioral and sleep-wake control. J. Neurosci..

[41] Huang Z.L., Mochizuki T., Qu W.M., Hong Z.Y., Watanabe T., Urade Y., Hayaishi O. (2006). Altered sleep-wake characteristics and lack of arousal response to H3 receptor antagonist in histamine H1 receptor knockout mice. Proc. Natl. Acad. Sci. USA.

[42] Gondard E., Anaclet C., Akaoka H., Guo R.X., Zhang M., Buda C., Franco P., Kotani H., Lin J.S. (2013). Enhanced histaminergic neurotransmission and sleep-wake alterations, a study in histamine H3-receptor knock-out mice. Neuropsychopharmacology.

[43] Thakkar M.M. (2011). Histamine in the regulation of wakefulness. Sleep Med. Rev..

[44] Monti J.M., Pellejero T., Jantos H. (1986). Effects of H1- and H2-histamine receptor agonists and antagonists on sleep and wakefulness in the rat. J. Neural. Transm..

[45] Lin J.S., Sakai K., Vanni-Mercier G., Arrang J.M., Garbarg M., Schwartz J.C., Jouvet M. (1990). Involvement of histaminergic neurons in arousal mechanisms demonstrated with H3-receptor ligands in the cat. Brain Res..

[46] Williams R.H., Chee M.J., Kroeger D., Ferrari L.L., Maratos-Flier E., Scammell T.E., Arrigoni E. (2014). Optogenetic-mediated release of histamine reveals distal and autoregulatory mechanisms for controlling arousal. J. Neurosci..

[47] Chung S., Weber F., Zhong P., Tan C.L., Nguyen T.N., Beier K., Hörmann N., Chang W.-C., Zhang Z., Do J.P. (2017). Identification of preoptic sleep neurons using retrograde labelling and gene profiling. Nature.

[48] Peyron C., Tighe D.K., Pol A.N.V.D., de Lecea L., Heller H.C., Sutcliffe J.G., Kilduff T. (1998). Neurons Containing Hypocretin (Orexin) Project to Multiple Neuronal Systems. J. Neurosci..

[49] Schöne C., Apergis-Schoute J., Sakurai T., Adamantidis A., Burdakov D. (2014). Coreleased Orexin and Glutamate Evoke Nonredundant Spike Outputs and Computations in Histamine Neurons. Cell. Rep..

[50] Huang Z.-L., Qu W.-M., Li W.-D., Mochizuki T., Eguchi N., Watanabe T., Urade Y., Hayaishi O. (2001). Arousal effect of orexin A depends on activation of the histaminergic system. Proc. Natl. Acad. Sci. USA.

[51] Sundvik M., Kudo H., Toivonen P., Rozov S., Chen Y., Panula P. (2011). The histaminergic system regulates wakefulness and orexin/hypocretin neuron development via histamine receptor H1 in zebrafish. FASEB J..

[52] Venner A., Mochizuki T., De Luca R., Anaclet C., Scammell T.E., Saper C.B., Arrigoni E., Fuller P.M. (2019). Reassessing the Role of Histaminergic Tuberomammillary Neurons in Arousal Control. J. Neurosci..

[53] Fujita A.A., Bonnavion P., Wilson M.M., Mickelsen L.L., Bloit J.J., De Lecea L.L., Jackson A.A. (2017). Hypothalamic Tuberomammillary Nucleus Neurons: Electrophysiological Diversity and Essential Role in Arousal Stability. J. Neurosci..

[54] Takeda N., Inagaki S., Shiosaka S., Taguchi Y., Oertel W.H., Tohyama M., Watanabe T., Wada H. (1984). Immunohistochemical evidence for the coexistence of histidine decarboxylase-like and glutamate decarboxylase-like immunoreactivities in nerve cells of the magnocellular nucleus of the posterior hypothalamus of rats. Proc. Natl. Acad. Sci. USA.

[55] Abdurakhmanova S., Grotell M., Kauhanen J., Linden A.M., Korpi E.R., Panula P. (2020). Increased Sensitivity of Mice Lacking Extrasynaptic δ-Containing GABA(A) Receptors to Histamine Receptor 3 Antagonists. Front. Pharmacol..

[56] Mickelsen L.E., Flynn W.F., Springer K., Wilson L., Beltrami E.J., Bolisetty M., Robson P., Jackson A.C. (2020). Cellular taxonomy and spatial organization of the murine ventral posterior hypothalamus. Elife.

[57] Vu M., Du G., Bayliss D.A., Horner R.L. (2015). TASK Channels on Basal Forebrain Cholinergic Neurons Modulate Electrocortical Signatures of Arousal by Histamine. J. Neurosci..

[58] Ericson H., Blomqvist A., Köhler C. (1989). Brainstem afferents to the tuberomammillary nucleus in the rat brain with special reference to monoaminergic innervation. J. Comp. Neurol..

[59] Scammell T.E. (2003). The neurobiology, diagnosis, and treatment of narcolepsy. Ann. Neurol..

[60] Nishino S., Sakurai E., Nevsimalova S., Yoshida Y., Watanabe T., Yanai K., Mignot E. (2009). Decreased CSF histamine in narcolepsy with and without low CSF hypocretin-1 in comparison to healthy controls. Sleep.

[61] Kanbayashi T., Kodama T., Kondo H., Satoh S., Inoue Y., Chiba S., Shimizu T., Nishino S. (2009). CSF histamine contents in narcolepsy, idiopathic hypersomnia and obstructive sleep apnea syndrome. Sleep.

[62] Bassetti C.L., Baumann C., Dauvilliers Y., Croyal M., Robert P., Schwartz J.-C. (2010). Cerebrospinal fluid histamine levels are decreased in patients with narcolepsy and excessive daytime sleepiness of other origin. J. Sleep Res..

[63] Franco P., Dauvilliers Y., Inocente C.O., Guyon A., Villanueva C., Raverot V., Plancoulaine S., Lin J. (2018). Impaired histaminergic neurotransmission in children with narcolepsy type 1. CNS Neurosci. Ther..

[64] John J., Thannickal T.C., McGregor R.M., Ramanathan L., Ohtsu H., Nishino S., Sakai N., Yamanaka A., Stone C., Cornford M. (2013). Greatly increased numbers of histamine cells in human narcolepsy with cataplexy. Ann. Neurol..

[65] Valko P.O., Gavrilov Y., Yamamoto M., Reddy H., Haybaeck J., Mignot E., Baumann C., Scammell T.E. (2013). Increase of histaminergic tuberomammillary neurons in narcolepsy. Ann. Neurol..

[66] Dauvilliers Y., Delallée N., Jaussent I., Scholz S., Bayard S., Croyal M., Schwartz J.-C., Robert P. (2012). Normal Cerebrospinal Fluid Histamine and tele-Methylhistamine Levels in Hypersomnia Conditions. Sleep.

[67] Croyal M., Dauvilliers Y., Labeeuw O., Capet M., Schwartz J.-C., Robert P. (2010). Histamine and tele-methylhistamine quantification in cerebrospinal fluid from narcoleptic subjects by liquid chromatography tandem mass spectrometry with precolumn derivatization. Anal. Biochem..

[68] Lamb Y.N. (2020). Pitolisant: A Review in Narcolepsy with or without Cataplexy. CNS Drugs.

[69] Dauvilliers Y., Arnulf I., Szakacs Z., Leu-Semenescu S., LeComte I., Scart-Gres C., LeComte J.-M., Schwartz J.-C., Bastuji H., Vieccherini M.F. (2019). Long-term use of pitolisant to treat patients with narcolepsy: Harmony III Study. Sleep.

[70] Lin J.S., Dauvilliers Y., Arnulf I., Bastuji H., Anaclet C., Parmentier R., Kocher L., Yanagisawa M., Lehert P., Ligneau X. (2008). An inverse agonist of the histamine H(3) receptor improves wakefulness in narcolepsy: Studies in orexin-/- mice and patients. Neurobiol. Dis..

[71] Owen M.J., Sawa A., Mortensen P.B. (2016). Schizophrenia. Lancet.

[72] Marder S.R., Cannon T.D. (2019). Schizophrenia. N. Engl. J. Med..

[73] McCutcheon R.A., Reis Marques T., Howes O.D. (2020). Schizophrenia-An Overview. JAMA Psychiatry.

[74] Prell G.D., Green J.P., Kaufmann C.A., Khandelwal J.K., Morrishow A.M., Kirch D.G., Linnoila M., Wyatt R.J. (1995). Histamine metabolites in cerebrospinal fluid of patients with chronic schizophrenia: Their relationships to levels of other aminergic transmitters and ratings of symptoms. Schizophr. Res..

[75] Jin C.Y., Anichtchik O., Panula P. (2009). Altered histamine H3 receptor radioligand binding in post-mortem brain samples from subjects with psychiatric diseases. Br. J. Pharmacol..

[76] Iwabuchi K., Ito C., Tashiro M., Kato M., Kano M., Itoh M., Iwata R., Matsuoka H., Sato M., Yanai K. (2005). Histamine H1 receptors in schizophrenic patients measured by positron emission tomography. Eur. Neuropsychopharmacol..

[77] Sato H., Ito C., Hiraoka K., Tashiro M., Shibuya K., Funaki Y., Yoshikawa T., Iwata R., Matsuoka H., Yanai K. (2015). Histamine H1 receptor occupancy by the new-generation antipsychotics olanzapine and quetiapine: A positron emission tomography study in healthy volunteers. Psychopharmacology.

[78] Kaminsky R., Moriarty T., Bodine J., Wolf D., Davidson M. (1990). Effect of famotidine on deficit symptoms of schizophrenia. Lancet.

[79] Mehta V.S., Ram D. (2014). Role of ranitidine in negative symptoms of schizophrenia-an open label study. Asian J. Psychiatr..

[80] Ligneau X., Landais L., Perrin D., Piriou J., Uguen M., Denis E., Robert P., Parmentier R., Anaclet C., Lin J.-S. (2007). Brain histamine and schizophrenia: Potential therapeutic applications of H3-receptor inverse agonists studied with BF2.649. Biochem. Pharmacol..

[81] Southam E., Cilia J., Gartlon J.E., Woolley M.L., Lacroix L.P., Jennings C.A., Cluderay J.E., Reavill C., Rourke C., Wilson D.M. (2008). Preclinical investigations into the antipsychotic potential of the novel histamine H3 receptor antagonist GSK207040. Psychopharmacology.

[82] Brown J.W., Whitehead C.A., Basso A.M., Rueter L.E., Zhang M. (2013). Preclinical evaluation of non-imidazole histamine H3 receptor antagonists in comparison to atypical antipsychotics for the treatment of cognitive deficits associated with schizophrenia. Int. J. Neuropsychopharmacol..

[83] Poyurovsky M., Fuchs C., Pashinian A., Levi A., Weizman R., Weizman A. (2012). Reducing antipsychotic-induced weight gain in schizophrenia: A double-blind placebo-controlled study of reboxetine–betahistine combination. Psychopharmacology.

[84] Barak N., Beck Y., Albeck J.H. (2016). A Randomized, Double-Blind, Placebo-Controlled Pilot Study of Betahistine to Counteract Olanzapine-Associated Weight Gain. J. Clin. Psychopharmacol..

[85] Fang Q., Hu W.-W., Wang X.-F., Yang Y., Lou G.-D., Jin M.-M., Yan H.-J., Zeng W.-Z., Shen Y., Zhang S.-H. (2013). Histamine up-regulates astrocytic glutamate transporter 1 and protects neurons against ischemic injury. Neuropharmacology.

[86] Liao R., Chen Y., Cheng L., Fan L., Chen H., Wan Y., You Y., Zheng Y., Jiang L., Chen Z. (2019). Histamine H1 Receptors in Neural Stem Cells Are Required for the Promotion of Neurogenesis Conferred by H3 Receptor Antagonism following Traumatic Brain Injury. Stem Cell Rep..

[87] Cheng L., Xu C., Wang L., An D., Jiang L., Zheng Y., Xu Y., Wang Y., Wang Y., Zhang K. (2021). Histamine H1 receptor deletion in cholinergic neurons induces sensorimotor gating ability deficit and social impairments in mice. Nat. Commun..

[88] Abeysinghe A., Deshapriya R., Udawatte C. (2020). Alzheimer’s disease; a review of the pathophysiological basis and therapeutic interventions. Life Sci..

[89] Vaz M., Silvestre S. (2020). Alzheimer’s disease: Recent treatment strategies. Eur. J. Pharmacol..

[90] Breijyeh Z., Karaman R. (2020). Comprehensive Review on Alzheimer’s Disease: Causes and Treatment. Molecules.

[91] Mazurkiewicz-Kwilecki I.M., Nsonwah S. (1989). Changes in the regional brain histamine and histidine levels in postmortem brains of Alzheimer patients. Can. J. Physiol. Pharmacol..

[92] Cacabelos R., Yamatodani A., Niigawa H., Hariguchi S., Tada K., Nishimura T., Wada H., Brandeis L., Pearson J. (1989). Brain histamine in Alzheimer’s disease. Methods Find Exp. Clin. Pharmacol..

[93] Panula P., Rinne J., Kuokkanen K., Eriksson K., Sallmen T., Kalimo H., Relja M. (1997). Neuronal histamine deficit in Alzheimer’s disease. Neuroscience.

[94] Shan L., Bossers K., Unmehopa U., Bao A.-M., Swaab D.F. (2012). Alterations in the histaminergic system in Alzheimer’s disease: A postmortem study. Neurobiol. Aging.

[95] Shan L., Swaab D.F., Bao A.-M. (2013). Neuronal histaminergic system in aging and age-related neurodegenerative disorders. Exp. Gerontol..

[96] Higuchi M., Yanai K., Okamura N., Meguro K., Arai H., Itoh M., Iwata R., Ido T., Watanabe T., Sasaki H. (2000). Histamine H1 receptors in patients with Alzheimer’s disease assessed by positron emission tomography. Neuroscience.

[97] Provensi G., Costa A., Izquierdo I., Blandina P., Passani M.B. (2018). Brain histamine modulates recognition memory: Possible implications in major cognitive disorders. Br. J. Pharmacol..

[98] Provensi G., Passani M.B., Costa A., Izquierdo I., Blandina P. (2018). Neuronal histamine and the memory of emotionally salient events. Br. J. Pharmacol..

[99] Dai H., Kaneko K., Kato H., Fujii S., Jing Y., Xu A., Sakurai E., Kato M., Okamura N., Kuramasu A. (2007). Selective cognitive dysfunction in mice lacking histamine H1 and H2 receptors. Neurosci. Res..

[100] Zlomuzica A., Ruocco L., Sadile A., Huston J., Dere E. (2009). Histamine H1 receptor knockout mice exhibit impaired spatial memory in the eight-arm radial maze. Br. J. Pharmacol..

[101] Zlomuzica A., Dere D., Dere E. (2013). The histamine H1 receptor and recollection-based discrimination in a temporal order memory task in the mouse. Pharmacol. Biochem. Behav..

[102] Dere E., Zlomuzica A., Viggiano D., Ruocco L., Watanabe T., Sadile A., Huston J., De Souza-Silva M. (2008). Episodic-like and procedural memory impairments in histamine H1 Receptor knockout mice coincide with changes in acetylcholine esterase activity in the hippocampus and dopamine turnover in the cerebellum. Neuroscience.

[103] Yanai K., Son L.Z., Endou M., Sakurai E., Nakagawasai O., Tadano T., Kisara K., Inoue I., Watanabe T. (1998). Behavioural characterization and amounts of brain monoamines and their metabolites in mice lacking histamine H1 receptors. Neuroscience.

[104] Schneider E.H., Neumann D., Seifert R. (2014). Modulation of behavior by the histaminergic system: Lessons from H1R-and H2R-deficient mice. Neurosci. Biobehav. Rev..

[105] Da Silveira C.K.B., Furini C.R., Benetti F., da Cruz Monteiro S., Izquierdo I. (2013). The role of histamine receptors in the consolidation of object recognition memory. Neurobiol. Learn Mem..

[106] Chen Z., Chen J.Q., Kamei C. (2001). Effect of H1-antagonists on spatial memory deficit evaluated by 8-arm radial maze in rats. Acta Pharmacol. Sin..

[107] Hasenöhrl R.U., Weth K., Huston J.P. (1999). Intraventricular infusion of the histamine H(1) receptor antagonist chlorpheniramine improves maze performance and has anxiolytic-like effects in aged hybrid Fischer 344xBrown Norway rats. Exp. Brain Res..

[108] Dere E., De Souza-Silva M.A., Topic B., Spieler R.E., Haas H.L., Huston J.P. (2003). Histidine-Decarboxylase Knockout Mice Show Deficient Nonreinforced Episodic Object Memory, Improved Negatively Reinforced Water-Maze Performance, and Increased Neo- and Ventro-Striatal Dopamine Turnover. Learn. Mem..

[109] Acevedo S.F., Ohtsu H., Benice T.S., Rizk-Jackson A., Raber J. (2006). Age-dependent measures of anxiety and cognition in male histidine decarboxylase knockout (Hdc-/-) mice. Brain Res..

[110] Liu L., Zhang S., Zhu Y., Fu Q., Zhu Y., Gong Y., Ohtsu H., Luo J., Wei E., Chen Z. (2007). Improved learning and memory of contextual fear conditioning and hippocampal CA1 long-term potentiation in histidine decarboxylase knock-out mice. Hippocampus.

[111] Gong Y.-X., Shou W.-T., Feng B., Zhang W.-P., Wang H.-J., Ohtsu H., Chen Z. (2010). Ameliorating effect of histamine on impairment of cued fear extinction induced by morphine withdrawal in histidine decarboxylase gene knockout mice. Acta Pharmacol. Sin..

[112] Schneider E.H., Neumann D., Seifert R. (2014). Modulation of behavior by the histaminergic system: Lessons from HDC-, H3R- and H4R-deficient mice. Neurosci. Biobehav. Rev..

[113] Rizk A., Curley J., Robertson J., Raber J. (2004). Anxiety and cognition in histamine H3 receptor-/- mice. Eur. J. Neurosci..

[114] Huang Y.-W., Hu W.-W., Chen Z., Zhang L.-S., Shen H.-Q., Timmerman H., Leurs R., Yanai K. (2004). Effect of the histamine H3-antagonist clobenpropit on spatial memory deficits induced by MK-801 as evaluated by radial maze in Sprague–Dawley rats. Behav. Brain Res..

[115] Orsetti M., Ghi P., Di Carlo G. (2001). Histamine H(3)-receptor antagonism improves memory retention and reverses the cognitive deficit induced by scopolamine in a two-trial place recognition task. Behav. Brain Res..

[116] Zlomuzica A., Dere D., Binder S., Silva M.A.D.S., Huston J.P., Dere E. (2015). Neuronal histamine and cognitive symptoms in Alzheimer’s disease. Neuropharmacology.

[117] Bardgett M.E., Davis N.N., Schultheis P.J., Griffith M.S. (2011). Ciproxifan, an H3 receptor antagonist, alleviates hyperactivity and cognitive deficits in the APP Tg2576 mouse model of Alzheimer’s disease. Neurobiol. Learn Mem..

[118] Nathan P.J., Boardley R., Scott N., Berges A., Maruff P., Sivananthan T., Upton N., Lowy M.T., Nestor P.J., Lai R. (2013). The safety, tolerability, pharmacokinetics and cognitive effects of GSK239512, a selective histamine H₃ receptor antagonist in patients with mild to moderate Alzheimer’s disease: A preliminary investigation. Curr. Alzheimer Res..

[119] Grove R.A., Harrington C.M., Mahler A., Beresford I., Maruff P., Lowy M.T., Nicholls A.P., Boardley R.L., Berges A.C., Nathan P.J. (2014). A randomized, double-blind, placebo-controlled, 16-week study of the H3 receptor antagonist, GSK239512 as a monotherapy in subjects with mild-to-moderate Alzheimer’s disease. Curr. Alzheimer Res..

[120] Haig G.M., Pritchett Y., Meier A., Othman A.A., Hall C., Gault L.M., Lenz R.A. (2014). A Randomized Study of H3 Antagonist ABT-288 in Mild-To-Moderate Alzheimer’s Dementia1. J. Alzheimer Dis..

[121] Egan M., Yaari R., Liu L., Ryan M., Peng Y., Lines C., Michelson D. (2012). Pilot randomized controlled study of a histamine receptor inverse agonist in the symptomatic treatment of AD. Curr. Alzheimer Res..

[122] Hallett M. (2015). Tourette Syndrome: Update. Brain Dev..

[123] Efron D., Dale R.C. (2018). Tics and Tourette syndrome. J. Paediatr. Child Health.

[124] Ercan-Sencicek A.G., Stillman A.A., Ghosh A.K., Bilguvar K., O’Roak B.J., Mason C.E., Abbott T., Gupta A., King R.A., Pauls D.L. (2010). L-Histidine Decarboxylase and Tourette’s Syndrome. N. Engl. J. Med..

[125] Fernandez T.V., Sanders S., Yurkiewicz I.R., Ercan-Sencicek A.G., Kim Y.-S., Fishman D.O., Raubeson M.J., Song Y., Yasuno K., Ho W.S. (2012). Rare Copy Number Variants in Tourette Syndrome Disrupt Genes in Histaminergic Pathways and Overlap with Autism. Biol. Psychiatry.

[126] Karagiannidis I., Dehning S., Sandor P., Tarnok Z., Rizzo R., Wolanczyk T., Madruga-Garrido M., Hebebrand J., Nöthen M., Lehmkuhl G. (2013). Support of the histaminergic hypothesis in Tourette Syndrome: Association of the histamine decarboxylase gene in a large sample of families. J. Med. Genet..

[127] Baldan L.C., Williams K.A., Gallezot J.-D., Pogorelov V., Rapanelli M., Crowley M., Anderson G.M., Loring E., Gorczyca R., Billingslea E. (2014). Histidine Decarboxylase Deficiency Causes Tourette Syndrome: Parallel Findings in Humans and Mice. Neuron.

[128] Rapanelli M., Frick L., Bito H., Pittenger C. (2017). Histamine modulation of the basal ganglia circuitry in the development of pathological grooming. Proc. Natl. Acad. Sci. USA.

[129] Rapanelli M., Frick L., Pogorelov V., Ohtsu H., Bito H., Pittenger C. (2017). Histamine H3R receptor activation in the dorsal striatum triggers stereotypies in a mouse model of tic disorders. Transl. Psychiatry.

[130] Raza C., Anjum R., Shakeel N.U.A. (2019). Parkinson’s disease: Mechanisms, translational models and management strategies. Life Sci..

[131] Radhakrishnan D.M., Goyal V. (2018). Parkinson’s disease: A review. Neurol. India.

[132] Braak H., Del Tredici K., Rüb U., de Vos R.A., Steur E.N.J., Braak E. (2003). Staging of brain pathology related to sporadic Parkinson’s disease. Neurobiol. Aging.

[133] Shan L., Liu C.-Q., Balesar R., Hofman M.A., Bao A.-M., Swaab D.F. (2012). Neuronal histamine production remains unaltered in Parkinson’s disease despite the accumulation of Lewy bodies and Lewy neurites in the tuberomamillary nucleus. Neurobiol. Aging.

[134] Nakamura S., Ohnishi K., Nishimura M., Suenaga T., Akiguchi I., Kimura J., Kimura T. (1996). Large neurons in the tuberomammillary nucleus in patients with Parkinson’s disease and multiple system atrophy. Neurology.

[135] Rinne J.O., Anichtchik O., Eriksson K.S., Kaslin J., Tuomisto L., Kalimo H., Röyttä M., Panula P. (2002). Increased brain histamine levels in Parkinson’s disease but not in multiple system atrophy. J. Neurochem..

[136] Anichtchik O., Rinne J.O., Kalimo H., Panula P. (2000). An Altered Histaminergic Innervation of the Substantia Nigra in Parkinson’s Disease. Exp. Neurol..

[137] Shan L., Bossers K., Luchetti S., Balesar R., Lethbridge N., Chazot P.L., Bao A.-M., Swaab D.F. (2012). Alterations in the histaminergic system in the substantia nigra and striatum of Parkinson’s patients: A postmortem study. Neurobiol. Aging.

[138] Liu C.-Q., Chen Z., Liu F.-X., Hu D.-N., Luo J.-H. (2007). Involvement of brain endogenous histamine in the degeneration of dopaminergic neurons in 6-hydroxydopamine-lesioned rats. Neuropharmacology.

[139] Liu C.-Q., Hu D.-N., Liu F.-X., Chen Z., Luo J.-H. (2008). Apomorphine-induced turning behavior in 6-hydroxydopamine lesioned rats is increased by histidine and decreased by histidine decarboxylase, histamine H1 and H2 receptor antagonists, and an H3 receptor agonist. Pharmacol. Biochem. Behav..

[140] Johnston T.H., Van Der Meij A., Brotchie J.M., Fox S.H. (2010). Effect of histamine H2 receptor antagonism on levodopa-induced dyskinesia in the MPTP-macaque model of Parkinson’s disease. Mov. Disord..

[141] Yang X., Cui G., Wang X., Yue X., Shi H., Shen X., Zhang Z. (2013). Ranitidine reduced levodopa-induced dyskinesia in a rat model of Parkinson’s disease. Neuropsychiatr. Dis. Treat..

[142] Nowak P., Noras Ł., Jochem J., Szkilnik R., Brus H., Körőssy E., Drab J., Kostrzewa R.M., Brus R. (2009). Histaminergic Activity in a Rodent Model of Parkinson’s Disease. Neurotox. Res..

[143] Masini D., Aguiar C.L., Bonito-Oliva A., Papadia D., Andersson R., Fisahn A., Fisone G. (2017). The histamine H3 receptor antagonist thioperamide rescues circadian rhythm and memory function in experimental parkinsonism. Transl. Psychiatry.

[144] Molinari S.P., Kaminski R., Rocco A., Yahr M.D. (1995). The use of famotidine in the treatment of Parkinson’s disease: A pilot study. J. Neural Transm..

[145] Schwartz J.-C. (2011). The histamine H3 receptor: From discovery to clinical trials with pitolisant. Br. J. Pharmacol..

